# Effect of excitatory and inhibitory agents and a glial inhibitor on optically-recorded primary-afferent excitation

**DOI:** 10.1186/1744-8069-4-39

**Published:** 2008-09-26

**Authors:** Hiroshi Ikeda, Takaki Kiritoshi, Kazuyuki Murase

**Affiliations:** 1 Department of Human and Artificial Intelligence Systems, Graduate School of Engineering, and Research and Education Program for Life Science, University of Fukui, 3-9-1 Bunkyo, Fukui 910-8507, Japan

## Abstract

The effects of GABA, excitatory amino-acid receptors antagonists and a glial metabolism inhibitor on primary-afferent excitation in the spinal dorsal horn were studied by imaging the presynaptic excitation of high-threshold afferents in cord slices from young rats with a voltage-sensitive dye. Primary afferent fibers and terminals were anterogradely labeled with a voltage-sensitive dye from the dorsal root attached to the spinal cord slice. Single-pulse stimulation of C fiber-activating strength to the dorsal root elicited compound action potential-like optical responses in the superficial dorsal horn. The evoked presynaptic excitation was increased by the GABA_A _receptor antagonists picrotoxin and bicuculline, by glutamate receptor antagonists D-AP5 and CNQX, and by the glial metabolism inhibitor mono-fluoroacetic acid (MFA). The increase in presynaptic excitation by picrotoxin was inhibited in the presence of D-AP5, CNQX and MFA. Presynaptic modulation in the central terminal of fine primary afferents by excitatory and inhibitory amino acids may represent a mechanism that regulates the transmission of pain.

## Introduction

The sensory information which arrives at the central terminals of sensory neurons in the spinal dorsal horn is regulated by presynaptic inhibition. The reduction in amplitude of propagated action potentials as a result of primary afferent depolarization (PAD) is thought to be a mechanism of presynaptic inhibition [for review, see [1]].

Early studies suggested that γ-aminobutyric acid (GABA) receptors at primary afferent terminals contribute to presynaptic inhibition. Pharmacological studies demonstrated a contribution of GABA_A _receptors to the induction of PAD in large primary afferents [for review see [2]], and that the GABA_A _receptor antagonists picrotoxin and bicuculline reduce PAD [3,4]. The possible presence of PAD in fine myelinated and unmyelinated primary afferent fibers has also been reported indirectly by measuring their antidromic activation thresholds [5-7], and by showing the depolarization of small-diameter dorsal root ganglion cells by GABA [8]. Recent studies report the possible contribution of excitatory amino-acid (EAA) receptors to PAD in fine primary afferent fibers by exogenous activation of presynaptic AMPA, kainite and NMDA receptors [9-11].

Although neurons neighboring afferent terminals had been thought to be a source of neurotransmitters which regulate presynaptic excitation, glial cells around afferent terminals have been proposed to be a source of these neurotransmitters [12,13]. It is reported that release of neurotransmitter from the presynaptic terminal not only stimulates the postsynaptic neuron but also activates the perisynaptic glial cells [13,14]. The activated glial cells, in turn, release neurotransmitters such as glutamate and/or ATP [12,14,15]. It is thought that these neurotransmitters can directly stimulate the postsynaptic neuron and can feed back onto the presynaptic terminal either to enhance or to depress further release of neurotransmitter [12,13].

Recently, we succeeded in recording the presynaptic excitation of fine afferents in a slice preparation of spinal dorsal horn by staining primary afferent fibers anterogradely from the dorsal root with a voltage-sensitive dye [16]. In the present study, using optical imaging along with various pharmacological agents, we examined the effects of glutamate receptors, GABA_A _receptor antagonists and a glial metabolic inhibitor on optically-recorded presynaptic excitation. Some of the results described here have been published in abstract form [17].

## Results

### Effect of picrotoxin on afferent-induced excitation in the dorsal horn

**Figure 1:**
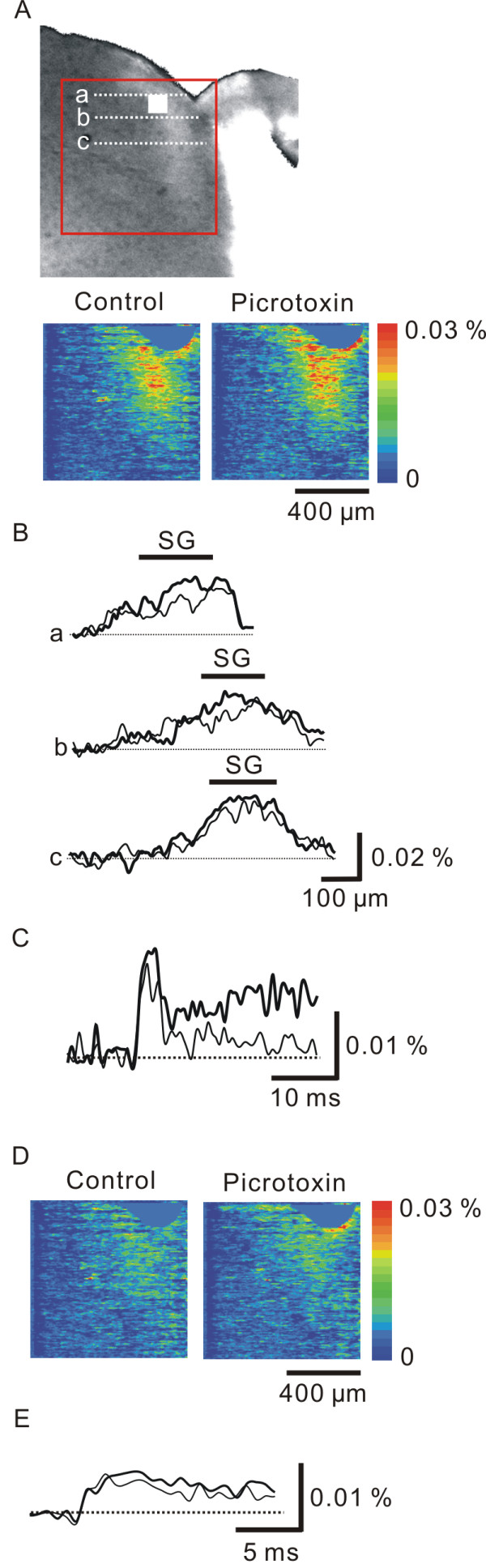
**Increase in net neuronal excitation in the spinal dorsal horn following application of picrotoxin.** A, Optical responses elicited by a high-intensity single-pulse stimulation (a current pulse of 2.0 mA with a duration of 0.5 ms) to the dorsal root in the control condition (left image) and in the presence of picrotoxin (right image) 4.8 ms after stimulation. Images were taken from the area indicated by the red square in the photo of the transverse slice. The percent change in light absorption is depicted using simulated color as described in the color bar. B, Spatial distributions of the optical responses in the control (thin lines) and picrotoxin (bold lines) conditions along three dorso-ventral lines, a-c, in the photo of a transverse slice indicated in A. Horizontal bars indicate the area of the substantia gelatinosa (SG) identified visually. C, Spatially averaged time courses of responses in control (thin line) and picrotoxin (bold line) conditions. The time courses were obtained in the dorsal horn from the area indicated by the filled white square in the photo of a transverse slice indicated in A. D, Optical responses elicited by a low-intensity single-pulse stimulation (a current pulse of 1.0 mA with a duration of 0.5 ms) to the dorsal root in control (left image) and picrotoxin (right image) conditions 4.8 ms after stimulation. Images were taken from the area indicated by the red square indicated in A. The percent change in the light absorption is depicted using simulated color as described in the color bar. E, Spatially averaged time courses of responses in control (thin line) and picrotoxin (thick line) conditions. The time courses were obtained in the dorsal horn from the area indicated by the filled white rectangle indicated in A.

Fig. 1A shows an example of optically recorded neuronal excitation elicited by high-intensity, single-pulse stimulation of the dorsal root (current pulse of 2.0 mA with a duration of 0.5 ms), which activates both the A and C fibers in the dorsal root, in a slice stained with a voltage-sensitive dye. As we have reported previously [16,18], dorsal-root stimulation induced prolonged neuronal excitation (> 100 ms) in lamina I-III of the spinal dorsal horn (Fig. 1C).

Bath application of a GABA_A _receptor antagonist, picrotoxin (100 μM), increased the optically-recorded net neuronal excitation, a sum of pre- and postsynaptic excitations (Fig. 1A–C, 129 ± 6%, p < 0.01, n = 9). The neuronal excitation induced by low-intensity stimulation (current pulse of 1.0 mA with a duration of 0.5 ms), which activates mainly A fibers in the dorsal root [18], was slightly increased by picrotoxin (Fig. 1D, E, 104 ± 5%, n = 5).

### The effect of capsaicin treatment

**Figure 2:**
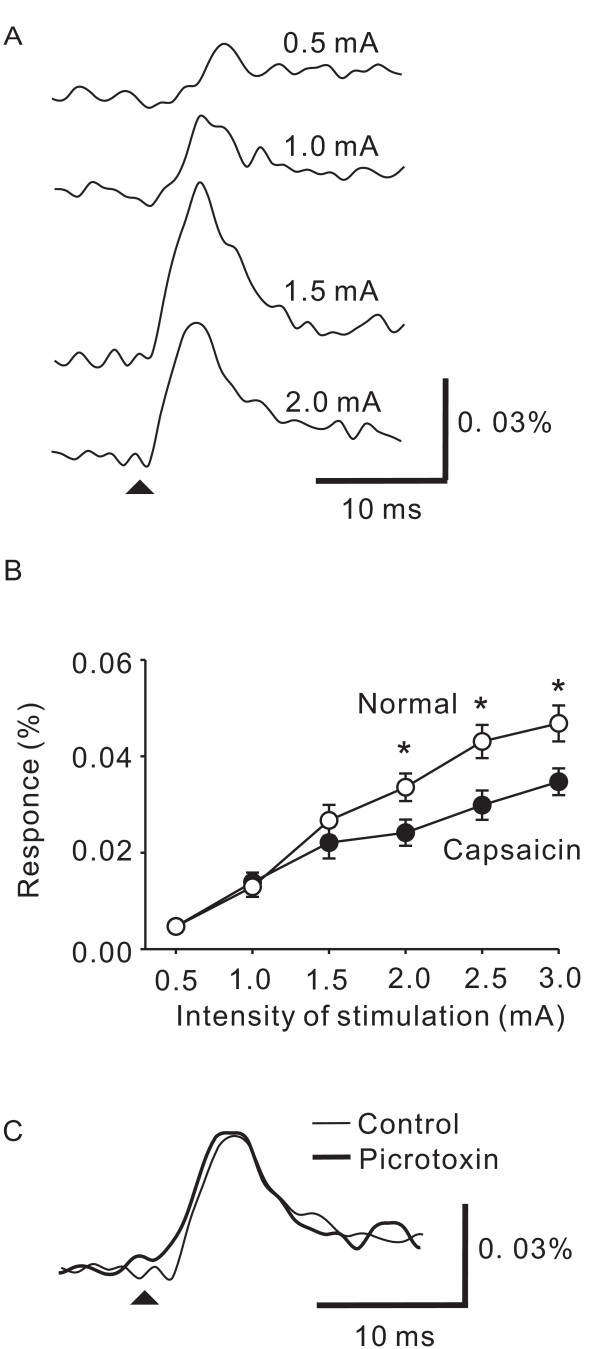
**The effect of picrotoxin on neuronal excitation in slices taken from capsaicin-treated rats.** A, Examples of optical response time courses in substantia gelatinosa of spinal dorsal horn elicited by various intensities of single-pulse stimulations to the dorsal root in a slice taken from a capsaicin-treated rat. The arrowhead indicates the time when stimulation was applied. B, The mean amplitude of optical responses elicited by the stimulation pulses shown in A in slices taken from normal rats (open circles) and capsaicin-treated rats (filled circles). C, Examples of time courses of optical responses in the substantia gelatinosa of the spinal dorsal horn elicited by a high-intensity single-pulse stimulation to the dorsal root in a slice taken from a capsaicin-treated rat in control (thin line) and picrotoxin (thick line) conditions. The arrowhead indicates the time when stimulation was applied. * P < 0.05.

To confirm that picrotoxin is effective on the neuronal excitation induced by C-fiber activity, we depleted most C-fiber inputs by neonatal capsaicin treatment [19] and then examined the effects of picrotoxin on the optical response. In slices taken from these capsaicin-treated rats, the magnitude of neuronal excitation evoked by high-intensity stimulation in the superficial dorsal horn was significantly smaller than that of normal rats (Fig. 2A, B, p < 0.05 at 2.0 mA, 2.5 mA, and 3 mA, n = 5). These results indicate that capsaicin-treated rats lacked capsaicin-sensitive C-fibers [20]. No significant potentiation was observed by picrotoxin in slices taken from the capsaicin-treated rats (Fig. 2C, 101 ± 3%, n = 9).

### Effect of picrotoxin on presynaptic excitation

**Figure 3:**
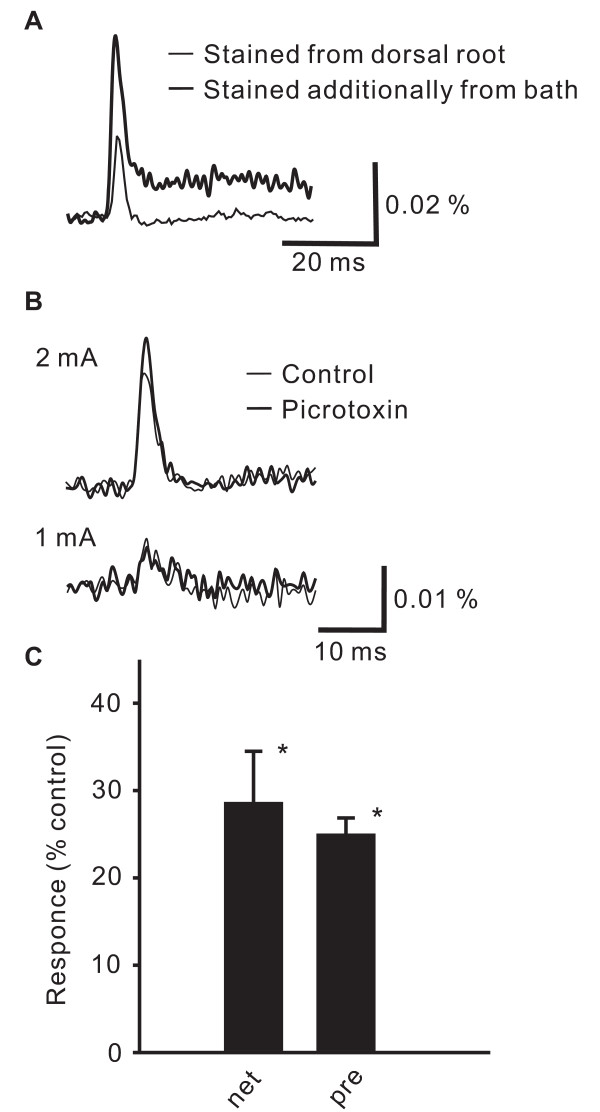
**The increase in presynaptic excitation in the spinal dorsal horn by picrotoxin.** A, Spatially averaged time course of the optical response elicited by a high-intensity single-pulse stimulation to the dorsal root in a slice stained anterogradely with a voltage-sensitive dye applied to the dorsal root (thin line), and the time course obtained from the same slice after bath application of the voltage-sensitive dye (thick line). These spatially averaged time courses were obtained in 16 × 16 pixels in the substantia gelatinosa. B, Spatially averaged time courses of optical responses in slices stained anterogradely with a voltage-sensitive dye. The upper and lower traces indicate the responses in the substantia gelatinosa of spinal dorsal horn by a high and a low-intensity stimulation in control (thin line) and picrotoxin (thick line) conditions, respectively. C, The bar graph summarizes the picrotoxin-induced increases in the net neuronal excitation (net), presynaptic excitation (pre).

We then stained only the primary afferent fibers for 3 hours via anterograde application of the voltage-sensitive dye in the suction pipette used for dorsal root stimulation. Dorsal root stimulation induced short-lasting (< 5 ms), action-potential or compound action potential-like optical signals in the spinal dorsal horn (thin trace in Fig. 3A) [16]. When the same slice was perfused with the solution containing the voltage-sensitive dye for 20 min, a prolonged component appeared in the optical response (bold trace in Fig. 3A), as was seen in the net excitation described in the previous section.

The presynaptic excitation induced by high-intensity stimulation, but not by low-intensity stimulation, was increased in the presence of picrotoxin (Fig. 3B &3C high-intensity: 125 ± 2%, p < 0.01, low-intensity: 83 ± 3%, n = 4). The presynaptic excitation induced by high-intensity stimulation was also increased in the presence of bicuculline (2 μM) (132 ± 7%, p < 0.01, n = 3).

### Effect of excitatory amino acid antagonists on presynaptic excitation

**Figure 4:**
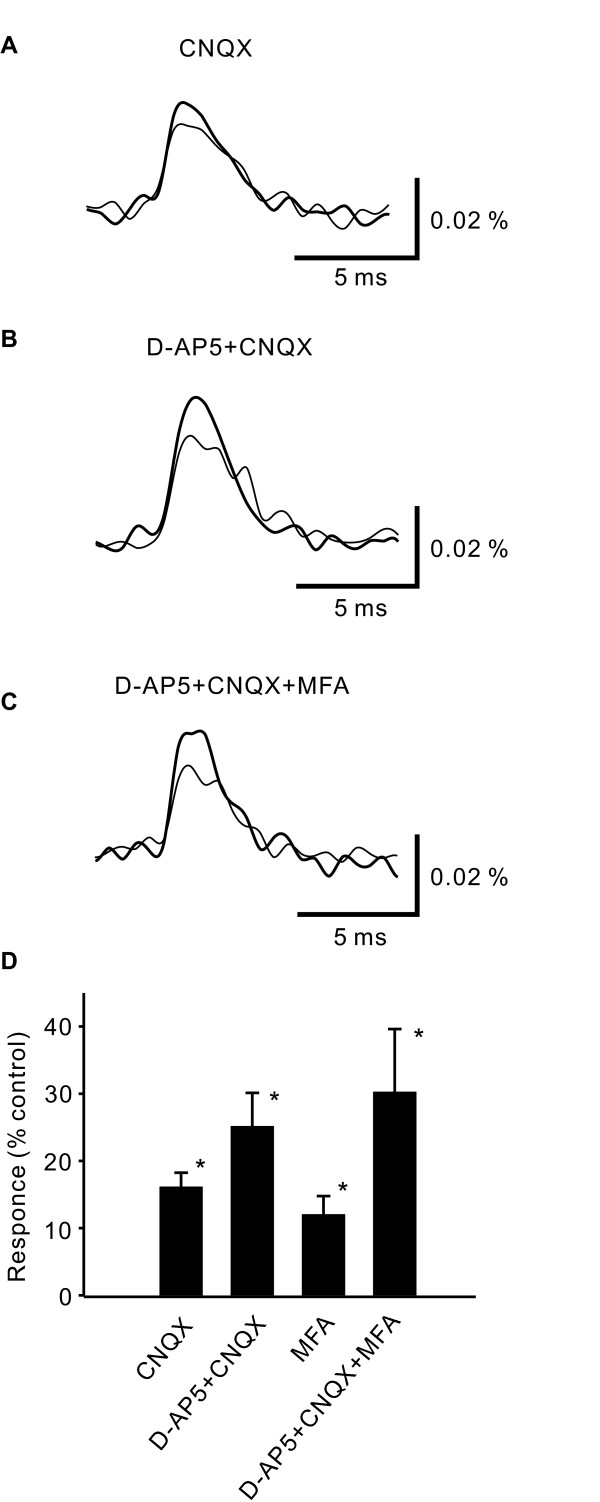
**The effect of glutamate receptor antagonists and a glial metabolism inhibitor on the increase of neuronal excitation by picrotoxin.** A, Spatially averaged time courses of presynaptic excitation in the substantia gelatinosa of spinal dorsal horn elicited by a high-intensity single-pulse stimulation to the dorsal root in the presence of D-AP5 and CNQX (thin line), and after adding picrotoxin (bold line). B, Spatially averaged time courses of the optical response in the substantia gelatinosa of spinal dorsal horn elicited by a high-intensity single-pulse stimulation to the dorsal root in the presence of D-AP5, CNQX and MFA (thin line), and after adding picrotoxin (bold line). C, The bar graph summarizes the picrotoxin-induced increases in the presynaptic excitation without D-AP5 and CNQX (w/o D-AP5+CNQX) in the presence of D-AP5 and CNQX (D-AP5+CNQX), and presynaptic excitation in the presence of D-AP5, CNQX and MFA (D-AP5+CNQX+MFA) elicited by high-intensity stimulation.

We next examined the effects of excitatory amino acid antagonists on presynaptic excitation induced by high-intensity stimulation. Application of the non-NMDA glutamate receptor antagonist 6-cyano-7-nitroquinoxaline-2,3-dione (CNQX, 10 μM) alone increased presynaptic excitation (Fig. 4A &4D, 116 ± 2%, p < 0.01, n = 4). Application of the NMDA-receptor antagonist D-2-amino-5-phosphonovaleric acid (D-AP5, 50 μM) together with CNQX produced a larger increase (Fig. 4B &4D, 125 ± 5%, p < 0.01, n = 4).

### Effect of picrotoxin in the presence of excitatory amino acid antagonists on presynaptic excitation

**Figure 5:**
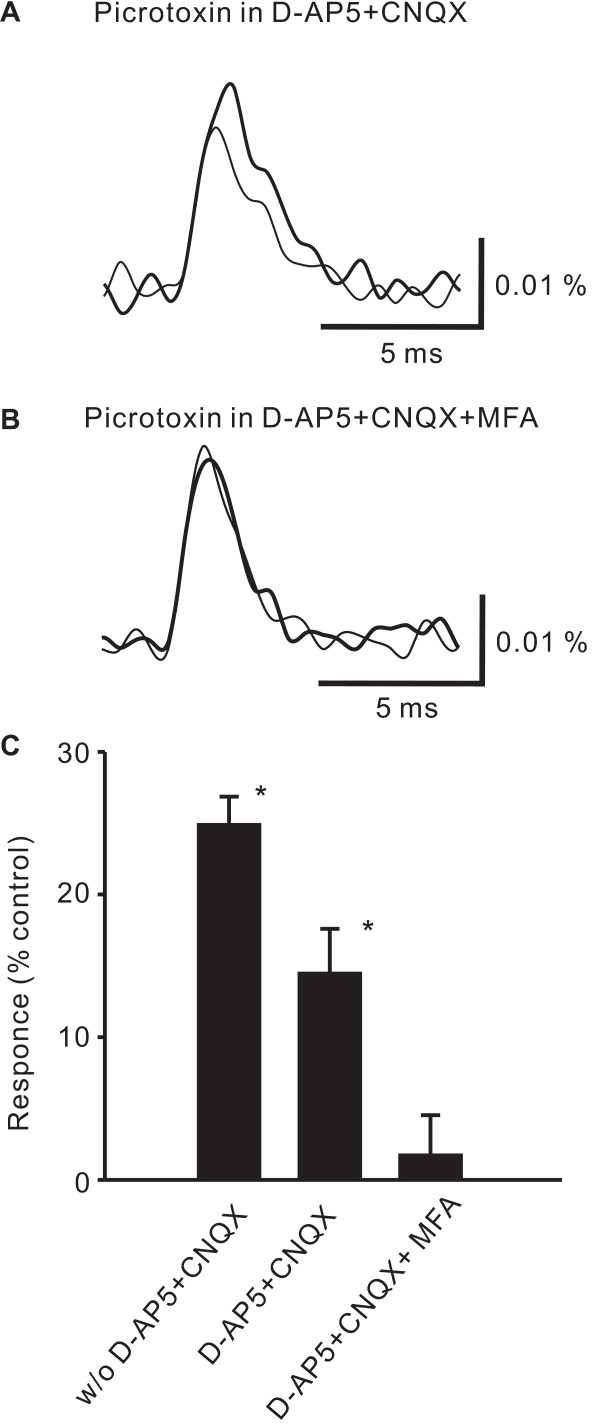
**The effect of glutamate receptor antagonists and a glial metabolism inhibitor on presynaptic excitation.** A, Spatially averaged time course of presynaptic excitation in the substantia gelatinosa in spinal dorsal horn elicited by high-intensity stimulation to the dorsal root in a control slice (thin line), and that after the application of CNQX (bold line). B, Spatially averaged time courses of presynaptic excitation before (thin) and after (thick) the application of D-AP5 and CNQX. C, Spatially averaged time courses of presynaptic excitation before (thin line) and after (bold line) the application of D-AP5 and CNQX and MFA. D, The bar graph summarizes the increases in presynaptic excitation induced by the application of CNQX, by D-AP5 and CNQX, and by D-AP5, CNQX, and MFA.

We further examined the effects of picrotoxin in the presence of excitatory amino acid antagonists on presynaptic excitation induced by high-intensity stimulation. In slices treated with both CNQX (10 μM) and D-AP5 (50 μM), the picrotoxin-induced increase in neuronal excitation was not augmented, but was smaller than that in control slices (Fig. 5A &5C, 114 ± 3%, p < 0.01, n = 4).

### Effect of glial metabolic inhibitor

Mono-fluoroacetic acid (MFA) is known to block glial metabolism [21]. Therefore, we next examined the effects of MFA on presynaptic excitation in the presence of EAA antagonists and picrotoxin.

MFA (5 mM) alone, and MFA together with D-AP5 and CNQX, increased the presynaptic excitation slightly (MFA alone: Fig. 4D, 112 ± 3%, p < 0.01, n = 3, MFA, D-AP5 & CNQX: Fig. 4C &4D, 130 ± 9%, p < 0.01, n = 4). MFA (5 mM), in addition to D-AP5 and CNQX, completely blocked the increase in neuronal excitation induced by picrotoxin (Fig. 5B &5C, 102 ± 3%, n = 4).

## Discussion

In this study, using optical imaging with a voltage-sensitive dye, we showed that net neuronal excitation evoked by dorsal root stimulation of C fiber-activating strength was potentiated by picrotoxin. We then recorded the excitation of only the presynaptic elements by anterograde staining via the dorsal root, and showed that it was also potentiated by picrotoxin and bicuculline. Application of CNQX alone potentiated the presynaptic excitation evoked by dorsal root stimulation. Application of CNQX and D-AP5 also potentiated the presynaptic excitation. The potentiation of presynaptic excitation by picrotoxin was inhibited by D-AP5 and CNQX. MFA alone potentiated slightly. Application of MFA together with D-AP5 and CNQX completely blocked the potentiation of presynaptic excitation by picrotoxin.

### Effect of picrotoxin on net neuronal excitation

Bath application of picrotoxin potentiated the net neuronal excitation in lamina I-III of the dorsal horn evoked by high-intensity dorsal root stimulation. We have previously shown that net excitation consists of early-presynaptic and delayed-postsynaptic components, and that the presynaptic excitation of A-fiber origin is much less than that of C-fiber origin [18]. In this study, in addition, we showed that the neuronal excitation elicited by high-intensity stimulation is weak in slices taken from neonatal capsaicin-treated rats that had lost their behavioral response to noxious stimulation, presumably due to the loss of their C-fibers [19]. Therefore, the neuronal excitation evoked by high-intensity stimulation mainly reflects the response to noxious stimuli. Under normal conditions, therefore, the nociceptive information in the superficial dorsal horn is persistently depressed via GABA_A _receptors.

Picrotoxin was more effective in neuronal excitation in slices taken from normal rats than from capsaicin-treated rats. These results suggest that the effects of picrotoxin observed in this study mainly reflect its effect on C-fibers. However, we can not separate the neuronal excitation induced by A-fibers from that by C-fibers only. We have shown that the neuronal excitation induced by the activation of large-diameter fibers is very small [18]. Therefore, we were unable to clarify whether or not the optically-recorded neuronal excitation induced by large-diameter fibers is potentiated by picrotoxin. There are many reports, in addition, demonstrating that GABA_A _receptors are expressed not only at central terminals of primary afferent fibers but also in dorsal horn neurons and that blocking GABA_A _receptors evokes excitation of dorsal horn neurons. Thus, it is expected that applying picrotoxin might also affect dorsal horn neuron excitability resulting from the blockade of GABA_A _receptors on dorsal horn neurons. Therefore, it is puzzling that the potentiation by picrotoxin was not observed in capsaicin-treated spinal cord slices.

### Effect of picrotoxin on presynaptic excitation

In this study, neuronal excitation of just the presynaptic elements was recorded by anterogradely staining with a voltage-sensitive dye applied via the dorsal root. This presynaptic excitation evoked by high-intensity dorsal root stimulation was not decreased by the application of the EAA antagonists, D-AP5 and CNQX. The anterograde staining, therefore, successfully labeled only presynaptic elements that consist of primary afferents and their terminals, but not postsynaptic neurons. Although it is impossible to measure the actual membrane potential values by the imaging system, it is highly likely that the evoked excitation represents compound action potentials in primary afferent fibers and/or terminals, because of its short duration.

Picrotoxin potentiated the evoked presynaptic excitation. This finding confirms that, under normal conditions, the generation of action potentials in primary afferents in the superficial dorsal horn is persistently inhibited via GABA_A _receptors.

### Effect of EAA antagonists on presynaptic excitation

The potentiation of presynaptic excitation was also observed by the application of EAA antagonists. It is reported that the receptors for EAA exist on primary afferent terminals, and that the activation of these receptors inhibits transmitter release from the terminals [9-11]. Therefore, the effect of EAA antagonists on presynaptic excitation may be due to the blockage of such EAA receptors on primary-afferent terminals. Alternatively, the action of EAA antagonists on postsynaptic GABAergic interneurons might have caused the EAA effect. In immunocytochemical studies, it was shown that GABAergic interneurons around primary afferent terminals make axoaxonic or dendroaxonic synapses in the superficial laminae of the dorsal horn [22-24]. In this study, the potentiation of presynaptic excitation by picrotoxin was not observed in the presence of D-AP5 and CNQX that inhibit excitatory synaptic transmission from primary afferents to postsynaptic neurons. These results favor the possibility that EAA antagonists inhibit the activity of GABAergic interneurons resulting in less release of GABA that acts on primary afferents. GABAergic interneurons thus might be the source for inhibition of presynaptic excitation, at least in part.

Since we applied glutamate receptor antagonists into the bath, activities of both postsynaptic excitatory neurons and GABAergic inhibitory neurons could be blocked unselectively, and there is no way to inhibit only one of the others, release of GABA and release of excitatory amino acids. It is therefore impossible to conclude which of the mechanisms takes the primary role at present. The possible inhibition via excitatory neurons, in addition to GABAergic inhibition, thus needs to be clarified in future with different techniques. It is also unclear whether the presynaptic inhibition observed in this study is due to the tonic neurotransmitter release or feedback responses from the activation of interneurons. The use of paired stimuli might provide the answer, by measuring the attenuation of the response to the second stimulus.

### Contribution of glial cells to presynaptic inhibition

The glial metabolism inhibitor MFA, together with the glutamate receptor antagonists D-AP5 and CNQX, completely blocked the picrotoxin-induced increase in presynaptic excitation. This result indicates that glial cells also contribute to GABAergic presynaptic inhibition. Several studies in culture [25,26] and in slices of the olfactory bulb [27] revealed that GABA or GABA-like substances can be secreted from glial cells, and they suggested that GABA released from glial cells is a source of tonic inhibition. Furthermore, in the spinal dorsal horn, it has been suggested that nociceptive transmission is inhibited by tonic GABA release [28,29]. Therefore, although there is no evidence that glial cells in the spinal dorsal horn can secret GABA, it is possible that nociceptive information in the spinal dorsal horn is inhibited by tonic release of GABA from glial cells. Thus, we still have to investigate whether the glial cells in the spinal dorsal horn can also produce and release GABA. In addition, because MFA may inhibit other glial functions, we also need to examine the effects of other glial functions on presynaptic inhibition. Experiments with other doses of MFA and/or other glial inhibitors are also necessary to confirm.

## Conclusion

**Figure 6:**
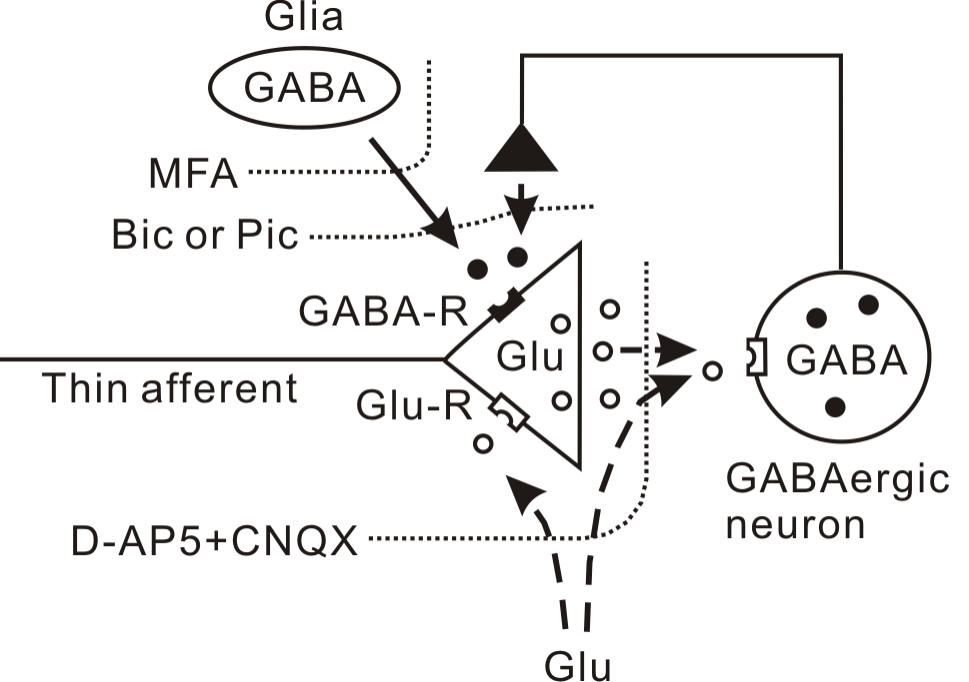
**Possible mechanism of presynaptic inhibition in primary afferent terminals by GABA.** Bath application of bicuculline (Bic) and/or picrotoxin (Pic) increases presynaptic excitation by blocking GABA receptors (GABA-R) on afferent terminals. MFA blocks the GABAergic action partially, inhibiting glial cells and leaving the neuronal path intact. Blocking glutamate receptors with D-AP5 and CNQX potentiates the presynaptic excitation by inhibiting the activity of GABAergic neurons and the presynaptic glutaminergic action. Further addition of MFA together with D-AP5 and CNQX removes all the inhibitory actions to the presynaptic terminals.

We demonstrated directly with an optical method that extracellular application of GABA receptor antagonists, glutamate receptor antagonists, and a glial metabolism inhibitor increases presynaptic excitation in the superficial dorsal horn. The increase in presynaptic excitation by picrotoxin was inhibited in the presence of glutamate receptor antagonists, and an inhibitor of glial metabolism. These results suggest that primary afferent terminals are inhibited by GABA release from GABAergic inhibitory neurons and also from glial cells (Fig. 6). Numerous reports suggest the inhibition of nociceptive information by GABA in the spinal dorsal horn. It is also reported that activation of GABA_A _receptors in the primary afferent terminals produces PAD [for review, see [2]], and that PAD reduces the amplitude of propagated action potentials in primary afferent terminals, thereby reducing neurotransmitter release [30]. In the processing of nociceptive information flow, PAD has been considered as a mechanism of the 'gate control' theory suggested by Melzack and Wall [31]. It is also suggested that PAD is a mechanism for allodynia and hyperalgesia in chronic pain [32]. Further studies may clearly confirm such pain control mechanisms by directly recording afferent excitation with an optical method, overcoming the difficulties encountered in previous studies.

## Methods

### Preparation

All animal studies were undertaken according to protocols approved by the university animal ethics committee. All efforts were made to minimize the number of animals used and their suffering. Eighteen- to 25-day-old Wister rats were anaesthetized by diethyl ether. Following laminectomy, the spinal cord was excised and several transverse slices (500 μm thick) with attached dorsal root were prepared from the lumbosacral enlargement. The animals were then sacrificed by an overdose of ether. Preparation for optical imaging of the gross neuronal excitation of afferent fibers has been described in detail [18]. In short, each slice was stained in a bath filled with the voltage-sensitive absorption dye, RH-482 (0.1 mg/ml, 20 min) and set in a submersion-type chamber (0.2 ml) on an inverted microscope (IMT, Olympus, Tokyo) equipped with a 150 W halogen lamp. A dorsal root of the slice was suctioned into a glass pipette from which stimulus current was applied. As we have reported [18], a single current pulse of 2 mA with a duration of 0.5 ms activated both A and C fibers evoking an intense optical signal in stained slices, while no signal was observed in unstained slices [33,34].

For optical imaging of excitation in primary afferent fibers, an unstained slice was set in the chamber, and the dorsal root was suctioned into a pipette filled with the voltage-sensitive dye (0.1 mg/ml) [16]. In response to the C-fiber-activating dorsal root stimulation, the optical response became observable after 3 hours of staining, but not within 1 to 2 hours. The staining period was thus fixed at 3 hours. The voltage-sensitive dye in the pipette was then washed out, and the dorsal root was re-suctioned into the pipette containing no dye.

Slices were perfused with Ringer's solution containing (in mM): 124 NaCl, 5 KCl, 1.2 KH_2_PO_4_, 1.3 MgSO_4_, 2.4 CaCl_2_, 26 NaHCO_3_, 0.2 thiourea, 0.2 ascorbic acid, and 10 glucose (oxygenated with 95% O_2 _and 5% CO_2_) at room temperature (23 ± 2°C). The same solution was used for the suction pipette.

### Optical recording

As described in Ikeda et al. [18], the light absorption change, at a wavelength of 700 ± 32 nm, in a 0.83 mm × 0.83 mm area of the dorsal horn was recorded by a Deltalon 1700 imaging system (Fuji Film Co., Tokyo) with 128 × 128 pixel photo sensors at a frame rate of 0.6 ms. The dorsal root was stimulated via a glass suction electrode. Eight single pulses were given at a constant interval of 15 s. Starting 10 ms before each stimulus, the image sensor took 128 consecutive frames of the light-absorption images at a sampling interval of 0.6 ms. A reference frame, which was taken immediately before each series of 128 frames, was subtracted from the subsequent frames. Eight series of such difference images were averaged and stored in the system memory. We determined the initial frame by averaging the first 15 frames of the difference image and then subtracting this average from each of the 128 frames of the image data on a pixel-by-pixel basis to eliminate the effects of noise contained in the reference frame. The ratio image was then calculated by dividing the image data by the reference frame.

As we have reported [33,34], by stimulation of 5 – 20 Hz for 1 s or longer to the dorsal root, a slow intrinsic optical signal with a duration of 1 – 2 minutes was elicited, but not by single-pulse stimulation. Therefore, the optical signals induced by single stimulation presented in this study primarily reflected the changes produced by the voltage-sensitive dye, presumably by the cellular electrical activities.

### Neonatal capsaicin treatment

Postnatal day 2 rats were anaesthetized with diethyl ether and injected subcutaneously at the dorsal cervix with a capsaicin solution (50 mg/kg). Three weeks after the injection, rats were tested for 1 min on a hot plate (65°C). While normal, untreated rats raised and licked their feet within 10 s, successfully treated rats did not react for at least 1 min.

### Drugs

The RH-482 (NK-3630) dye was obtained from Nippon Kanko Shikiso (Okayama, Japan). The D-2-amino-5-phosphonovaleric acid, capsaicin, and monofluoroacetic acid, were from Sigma (St. Louis, MO). 6-cyano-7-nitroquinoxaline-2,3-dione, picrotoxin and bicuculline methochloride were from Tocris Cookson Ltd. (Bristol, UK).

### Statistics

Results were expressed as means ± SE. Paired Student's *t*-tests or non-parametric ANOVA (Tukey-Kramer test) were used to examine statistical differences.

## Competing interests

The authors declare that they have no competing interests.

## Authors' contributions

HI and KM participated in the conception, design, and interpretation of the study. HI carried out the experiments, performed the data analysis. All authors wrote the manuscript.
